# Weight Cycling Increases Longevity Compared with Sustained Obesity in Mice

**DOI:** 10.1002/oby.22290

**Published:** 2018-10-25

**Authors:** Daniel L. Smith, Yongbin Yang, Tim R. Nagy, Amit Patki, Joseph R. Vasselli, Yiying Zhang, Stephanie L. Dickinson, David B. Allison

**Affiliations:** ^1^ Department of Nutrition Sciences University of Alabama at Birmingham Birmingham Alabama USA; ^2^ Nutrition Obesity Research Center University of Alabama at Birmingham Birmingham Alabama USA; ^3^ Diabetes Research Center University of Alabama at Birmingham Birmingham Alabama USA; ^4^ Nathan Shock Center of Excellence in the Basic Biology of Aging University of Alabama at Birmingham Birmingham Alabama USA; ^5^ Department of Biostatistics University of Alabama at Birmingham Birmingham Alabama USA; ^6^ Department of Medicine, New York Obesity‐Nutrition Research Center, College of Physicians and Surgeons Columbia University New York New York USA; ^7^ Division of Molecular Genetics, Department of Pediatrics, New York Obesity‐Nutrition Research Center, College of Physicians and Surgeons Columbia University New York New York USA; ^8^ Department of Statistics and Department of Epidemiology and Biostatistics Indiana University Bloomington Indiana USA; ^9^ Office of Energetics, School of Health Professions University of Alabama at Birmingham Birmingham Alabama USA

## Abstract

**Objective:**

Despite the known health benefits of weight loss among persons with obesity, observational studies have reported that cycles of weight loss and regain, or weight cycling, are associated with increased mortality. To study whether weight loss must be sustained to achieve health and longevity benefits, we performed a randomized controlled feeding study of weight cycling in mice.

**Methods:**

In early adult life, obese mice were randomized to *ad libitum* feeding to sustain obesity, calorie restriction to achieve a “normal” or intermediate body weight, or weight cycling (repeated episodes of calorie restriction and *ad libitum *refeeding). Body weight, body composition, and food intake were followed longitudinally until death. A subsample of mice was collected from each group for determination of adipose cell size, serum analytes, and gene expression.

**Results:**

Weight loss significantly reduced adipose mass and adipocyte size in both sexes, whereas weight cycling animals regained body fat and cell size during refeeding. Sustained weight loss resulted in a dose‐dependent decrease in mortality compared with *ad libitum *feeding.

**Conclusions:**

Weight cycling significantly increased life‐span relative to remaining with obesity and had a similar benefit to sustained modest weight loss.

## Abstract 2

**See Commentary**, pg. https://doi.org/10.1002/oby.22335.

## Introduction

Obesity has substantially increased in prevalence in modern times and is associated with both chronic health conditions (e.g., type 2 diabetes, cardiovascular disease, cancer) and increased mortality rate (1, 2). Although intentional weight loss improves the acute risk for noncommunicable diseases and biomarkers of health (3, 4), the effect of weight loss on longevity as assessed by observational studies remains disputed (5, 6, 7, 8). This may be particularly so given the difficulty in maintaining achieved weight loss for extended durations of time, resulting in subsequent unintentional weight regain. This pattern is often repeated as a recurring loss‐regain cycle referred to as weight cycling or “yo‐yo dieting” (9, 10). Relationships between weight change and mortality may be further obfuscated by attempts to assess whether the weight loss was intentional or unintentional, such as that resulting from occult disease (11, 12). Even the expression of intention for weight loss alone may not be sufficient to remove this confounding (13). These difficulties with weight loss and uncertainties concerning benefits for longevity have left some questioning whether weight loss is even worth the effort (9). To more directly study the associations between weight change and mortality, we used an established murine model to perform a randomized controlled feeding study with more than 900 individually housed mice to assess whether repeated bouts of weight loss by calorie (energy) restriction (CR) and weight regain by *ad libitum* feeding (weight cycling) in overweight or obese rodents would alter survival relative to maintained obesity (*ad libitum *feeding) or weight reduction (sustained CR).

## Methods

### Animals

C57BL/6J male and female mice were purchased from the Jackson Laboratory (Bar Harbor, Maine) at 6 weeks of age and were acclimated to the specific‐pathogen‐free facility for 2 weeks. All mice were singly housed for the duration of the study in standard, ventilated mouse cages within a Thoren Rack Mobile Housing System (Thoren Caging Systems, Inc., Hazleton, Pennsylvania) as described (14, 15). Animal rooms were maintained at 20°C to 22°C on a 12‐hour light‐dark cycle from 6:00 am to 6:00 pm. Animal health was checked daily, and moribund animals were euthanized according to the study protocol. All study protocols were approved by the University of Alabama at Birmingham Institutional Animal Care and Use Committee. Some data from this study have been described elsewhere (15).

### Study design

Beginning at 8 weeks of age, the C57BL/6J male and female mice were provided free access to a high‐fat diet (45% kcal fat and 20% protein based on D12451 [calorie: 4.73 kcal/g]; Research Diets, New Brunswick, New Jersey) (Supporting Information Table S1) to determine *ad libitum* intake. At 10 months of age, the mice were weighed, and the heaviest two‐thirds of the mice were subsequently randomized by quartile of body weight within each sex into diet groups (continuing with the high‐fat diet feeding until death). In order to assess the impact of weight loss and cycling from a state of excess body weight, the other one‐third of mice, with the lowest body weight, were removed from the study, as they were naturally resistant to diet‐induced obesity. The four diet groups were as follows: the Ever Obese (EO) group, which continued *ad libitum* feeding; the Obese Weight Loss (OWL) group, in which energy intake was restricted by nearly 30% of EO intake (with the vitamin and mineral mix supplemented in the high‐fat diet when restriction was > 20%; Research Diets D11022101) (Supporting Information Table S1); the Obese Weight Loss Moderate (OWLM) group, in which energy intake was restricted by nearly 20% of EO intake; and the Weight Cyclers (WC) group, in which energy restriction was enforced by dietary restriction followed by subsequent periods of *ad libitum *refeeding. The OWL group was designed to reduce the body weight of the mice to a weight comparable with that of C57BL/6J male and female mice (*n* = 15/sex) fed a low‐fat diet (10% kcal fat) at our facility (Supporting Information Table S1 and Table S10). This low‐fat‐fed (sex‐specific) body weight was also used as a target mean body weight for the WC group during periods of energy restriction (which was applied through a gradual, stepped restriction ~10% intervals). Periods of weight loss (~3‐month periods) and regain (~4‐month periods) were repeated as sequential intervals for the remaining life of the WC group following randomization. The OWLM group was designed to reduce the body weight of the mice to approximately the midpoint of the EO and OWL groups. Because of the size of the study, the total sample was divided and performed in two waves, and the two cohorts of animals were separated by approximately one calendar year. Experimental wave 1 included both male and female mice, whereas wave 2 contained only male mice because of a higher than expected incidence of ulcerative dermatitis early in life for the females in experimental wave 1. Additionally, the vitamin A levels in the high‐fat diet for experimental wave 2 were reduced to the levels recommended by the National Research Council (1995), with the same high‐fat diet formulation (Research Diets number D11112301) (Supporting Information Table S1), as vitamin A had been implicated in ulcerative dermatitis development (16). Following randomization, weekly food intake was measured for animals provided *ad libitum* access (e.g., EO group) along with weekly body weights for all animals. For animals receiving a daily allotment of a restricted amount of food (OWL and OWLM groups), fresh allotments were provided approximately 1 to 2 hours before lights off, and any food remaining after 24 hours was recorded and discarded before the next day’s provisions were given.

### Life‐span

For the C57BL/6J mice, observed life‐span (days) was recorded as the age when the animals died naturally or were euthanized owing to morbidity. Natural death or moribund status termination was recorded to the nearest day. Causes of death were categorized as (1) euthanized for ulcerative dermatitis or similar skin lesions (*n* = 290), (2) euthanized for other reasons, such as disability to eat or drink (*n* = 42), (3) found dead (*n* = 211), and (4) died from technical complications related to procedures (e.g., anesthesia, physical cage injury, veterinary treatment [*n* = 9]). Sex and experimental waves of each individual were adjusted when comparing life‐span among treatment assignments.

### Body composition

From 47 weeks of age, body composition (fat and fat‐free mass) was determined *in vivo* in all groups by quantitative magnetic resonance (EchoMRI 3‐in‐1, V2.1; Echo Medical Systems, Houston, Texas) at peak and trough points of the WC group (~3‐4 month intervals). The specific week of measurement differed between male and female mice to allow the time needed to reach the targeted weight or to stagger measurements to accommodate the number of animals.

### Dissection and tissue collection

Following the diet group randomization and initial weight loss phase, wherein mean body weight of OWL and WC was similar to low‐fat‐fed reference animals (~13 months of age), subsamples of mice from each of the various diet groups (*n* = 5/sex/group) were euthanized by decapitation for blood and tissue collection (e.g., adipose depots, skeletal muscle, liver, heart), with an additional sample of 5/sex from the WC group following refeeding and weight regain. Tissues were weighed to the nearest 0.01 g and either fixed or quickly frozen in screw‐capped microtubes in liquid nitrogen and stored at −80°C until use. Again, at approximately 23 months of age (following the ~50% mortality point), random subsamples of mice remaining alive from all groups were collected as described, with an additional 5/sex in the WC group. All dissections were performed with wave 1 animals during the light phase and by the same individuals, who were blinded to group assignment.

### Fat cell size and number

Samples of inguinal and gonadal white adipose tissue were collected from the subgroups (*n* = 5/sex/group) at the specified time points above. Cell size and number were measured by using osmium fixation technique as described previously, with ~100 mg of right inguinal and gonadal adipose tissue weighed to the nearest 0.01 g (17, 18).

### Serum analytes

Total blood was collected from the trunk by decapitation and kept on ice for at least 15 minutes. Samples were centrifuged for 10 minutes at 3,000*g* at 4°C, with the serum layer carefully collected. Serum glucose was measured by using an Analox GM7 analyzer (Analox Instruments, Lunenburg, Massachusetts), serum insulin by using Millipore Sensitive Rat Insulin Radioimmunoassay (RIA) (Millipore, Billerica, Massachusetts), serum adiponectin and leptin with Millipore enzyme‐linked immunosorbent assay (ELISA) kits, and proinflammatory profiles with the V‐PLEX Panel 1 kit (Meso Scale Diagnostics, Rockville, Maryland).

### Gene expression

Quantitative real‐time polymerase chain reaction (PCR) was used to determine gene expression as previously described (19). Briefly, total RNA was isolated using RNeasy mini‐columns (Qiagen, Inc., Valencia, California) and reverse‐transcribed into single‐stranded complementary DNA (cDNA) using random hexamers and M‐MLV Reverse Transcriptase (Invitrogen, Carlsbad, California). Quantitative amplification of cDNA of interest by PCR was carried out using gene‐specific primers and iQ SYBR Green Supermix (Bio‐Rad Laboratories, Hercules, California). To prevent the amplification of any contaminating genomic DNA, the forward and reverse PCR primers were derived from two different exons that are separated by at least a 1‐kilobase intron. The size of the amplicon was confirmed by agarose gel electrophoresis. Cyclophilin A mRNA level was used to normalize total RNA input. The difference in PCR cycle numbers at the specified fluorescence thresholds (within the linear amplification range) for the gene of interest and cyclophilin A (delta Ct) was used to calculate the mRNA level of the gene of interest. All quantitative real‐time PCRs were performed in triplicate, and the arithmetic mean of the triplicate was used in subsequent calculations.

### Statistical analysis

Power estimates were performed using the primary outcome of survival for male and female C57BL/6J mice from published survival data. Calculated samples sizes were estimated to provide ≥ 80% to reject the null hypothesis of no difference in survival for each group pairwise comparison with no intentional weight loss (EO group). Statistical analyses were performed using SAS software (version 9.4 for Windows, SAS Institute, Cary, North Carolina). The mean life‐spans of the three CR groups were compared with the mean life‐span of the EO group using survival analysis by running the Cox proportional hazard regression model. Hazard ratios were estimated between each CR group and the EO group. Primary survival analyses were performed for all‐cause mortality (as an intent‐to‐treat model with no censoring) as well as with accommodations by censoring for type of death (e.g., technical complications, technical complications and euthanized for reasons other than ulcerative dermatitis, found dead) (Supporting Information Table S7). Our study was designed to last until all the animals died, permitting us to also run generalized linear models to compare the mean life‐spans of the CR groups with the mean life‐span of the EO group.

### Mediation analysis

In investigating the effect on life‐span of groups created on the basis of CR, we also hypothesized that there could be a clinical variable acting as a mediator. Food efficiency, percent body fat, and fat to lean ratio were tested for mediation effect. Given known concerns with the use of ratios, additional sensitivity analyses were performed (data not shown) using covariates in the analyses as previously described (20). Because the WC group animals were subjected to increase and decrease their body weights, the mediation analysis was done only between specific time points, during which the animals in the WC group were in either the weight gain (week 57 to week 73) or the weight loss (week 73 to week 85) phase. Sobel’s test was performed to test the significance of mediation effect in predicting life‐span (21).

The differences in levels of serum cytokines and mRNA expression among the EO and CR groups were tested by using analysis of covariance (ANCOVA) (adjusting for sex) within the inguinal and gonadal adipose depots at four time points.

### Data availability

Data pertaining to the findings presented in this article will be made publicly available through a repository.

## Results

### Body weight and adiposity

**Figure 1:**
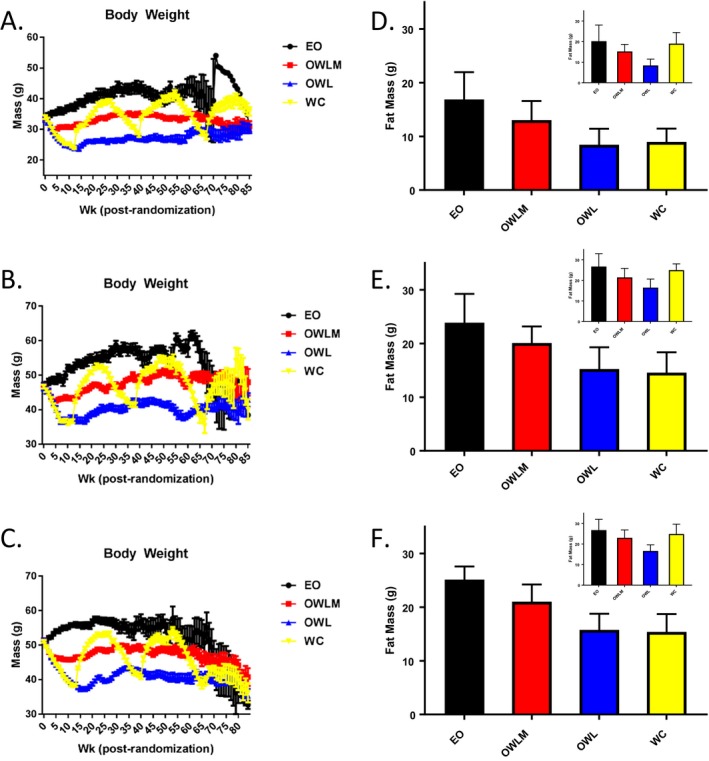
**Body weight and body composition.** Weekly body weight after randomization (mean ± SE) by group, including (**A**) wave 1 female, (**B**) wave 1 male, and (**C**) wave 2 male (all waves and sexes *P* < 0.01 [OWL, OWLM, and WC] vs. EO in postrandomization weeks 12‐50). A small sample (*n* = 15/sex) of low‐fat‐fed mice were used as a “benchmark” for the OWL body weight target (see Supporting Information Table S10. Body fat mass as measured by quantitative magnetic resonance (mean ± SD) following initial weight loss (T1, ~week 13; *P* < 0.01 all groups vs. EO) and regain by the WC group (T2, ~week 29; EO vs. WC, *P*  not significant). (**D**) wave 1 female; (**E**) wave 1 male; (**F**) wave 2 male. EO, Ever Obese (black); OWLM, Obese Weight Loss Moderate (red); OWL, Obese Weight Loss (blue); QMR, Quantitive Magnetic Resonance; WC, Weight Cyclers (yellow).

Because we wished to test the effect of weight loss and regain from an overweight or obesity state that represented a dietary model of obesity rather than a specific genetic condition, we used a high‐fat diet (45% of kilocalories from fat) feeding paradigm that successfully induced obesity in both sexes of mice by 10 months of age relative to animals fed a low‐fat diet (14). Furthermore, individuals who diet to lose body weight represent a population with unwanted excess body weight, particularly body fat. To more accurately model this physiologic condition, animals that were more naturally resistant to high‐fat diet‐induced obesity were removed from the study at 10 months of age, leaving the top two‐thirds of the heaviest animals for group randomization to continued *ad libitum* high‐fat feeding or one of three CR paradigms, with diet macronutrient composition held constant (Supporting Information Table S1). Following group randomization, CR was instituted by gradual stepped food restriction, resulting in significant reductions in body weight (Figure 1A‐1C), body fat, and lean mass at the whole‐animal level (Figure 1B‐1D; Supporting Information Figure S1A‐I). These body composition changes were further reflected as lower individual adipose depot masses among CR animals (vs. EO controls) after initial weight loss and at approximately 2 years of age (Supporting Information Figure S2A‐D; Supporting Information Table S2). Furthermore, this adipose reduction was accompanied by decreased inguinal (subcutaneous) and gonadal (visceral) white adipocyte cell size with initial restriction relative to the EO groups (Figure S3A‐S3H). Each aspect of body composition rebounded after release from CR to *ad libitum* refeeding, with subsequent weight regain for WC animals, including body weight, body fat, lean mass, and adipocyte cell size, as well as after sequential weight cycling episodes (Figure 1A‐1C; Supporting Information Figure S1‐S3).

### Hormones and glucose

Coincident with the changes in body weight, fat mass, and cell size, circulating leptin levels were significantly different among groups after initial weight loss and at the midpoint of life‐span (Supporting Information Table S3), in agreement with adipose tissue expression levels (Supporting Information Table S4). There were no significant differences among diet groups detected for circulating glucose and adiponectin after initial weight loss or the midpoint of life‐span (Supporting Information Table S3), in contrast with adiponectin expression from adipose depots at midlife (Supporting Information Table S4). Insulin levels were significantly different after initial weight loss (lower in each of the diet groups compared with EO) (Supporting Information Table S3). Additionally, there were significant differences between sexes in glucose, insulin, adiponectin, and leptin after initial weight loss, with only adiponectin remaining significant at the midpoint of survival (Supporting Information Table S3).

### Longevity

**Figure 2:**
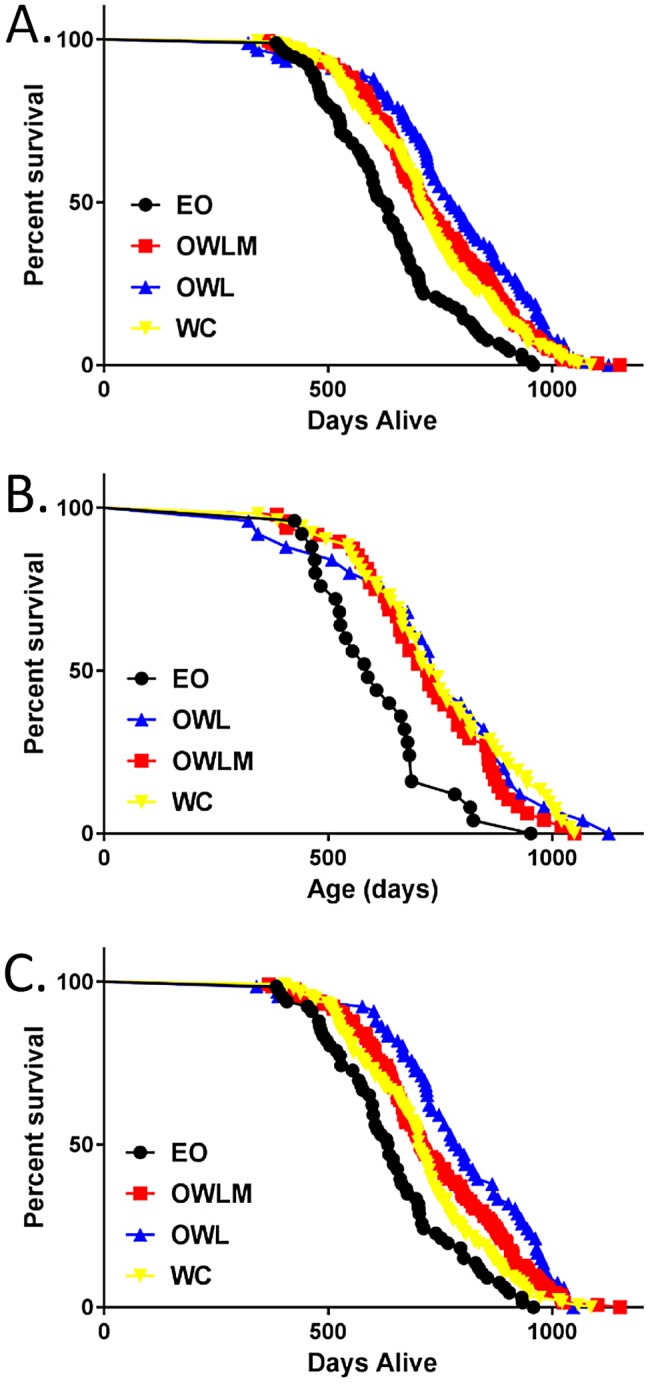
**Kaplan Meier Survival Plot. Survival distribution by days alive in the study by treatment.** (**A**) Wave and sex combined, (**B**) female only, and (**C**) male only survival. EO, Ever Obese (black; *n* = 25 female, 66 male); OWLM, Obese Weight Loss Moderate (red; *n* = 48 female, 132 male); OWL, Obese Weight Loss (blue; *n* = 26 female, 66 male); WC, Weight Cyclers (yellow; *n* = 51 female, 138 male).

We next asked whether mortality rate was altered in the various diet groups. CR resulted in a significant reduction in mortality rate relative to the *ad libitum* (EO) control for both males and females (*P* < 0.001 for all groups vs. EO) (Figure 2; Table 1; Supporting Information Table S5 and S6). Furthermore, WC mice were significantly longer lived than their *ad libitum* EO counterparts for mean, median, and maximum life‐span in both sexes (Figure 2; Table 1; Supporting Information Table S5 and S6), albeit not as long lived as the group that achieved and maintained a “normal” body weight by continual CR (OWL, *P* = 0.001; Figure 2; Table 1; Supporting Information Table S5). The improved longevity of the WC group, as well as the OWL and OWLM groups relative to the EO group, was present in both males and females, regardless of the cause of death (e.g., died spontaneously, euthanized for moribund conditions, combined all‐cause mortality) (Supporting Information Table S7). Additionally, for overall mortality risk, the mortality of the WC group was not significantly different from losing and maintaining an intermediate amount of weight (OWLM group) over the life‐span (*P* = 0.28; Figure 2; Table 1; Supporting Information Table S5 and S6).

**Table 1:** Survival analyses: general linear model and Cox proportional hazard

**Data**	**Predictor**		**Linear model**			**Survival model (Cox PH)**	
			***F* stat/*t*** a **stat**	***P***		**HR**	***P***
**Wave 1 and 2**	**Group**		13.02	<0.001			<0.001
	**EO vs. OWL**		6.07^a^	<0.001^b^		0.38	<0.001
	**EO vs. OWLM**		4.68^a^	<0.001^b^		0.51	<0.001
	**EO vs. WC**		3.85^a^	<0.001^b^		0.57	<0.001
	**OWLM vs. WC**		1.07^a^	0.868^b^		0.89	0.253

Using all animals without censoring.

a *t* statistic.

b Corrected for multiple comparison using Sidak.

### Mediators

Energy restriction and excessive weight reduction result in multiple physiologic, health, and longevity effects associated with the amount and duration of restriction. Dietary, genetic, and environmental interventions that alter growth and body size within a species further support an inverse relationship between body size and longevity (i.e., smaller mice living longer than larger mice). To assess this relationship between body size and longevity, we assessed animal‐specific age at maximum body weight in relationship to longevity. Similar to previous reports, there was a significant positive correlation between age at maximum body weight and longevity across diet groups; mice that grew more slowly lived longer (Supporting Information Table S8). To integrate energy intake with energy storage data (body weight gain, incorporating body composition), we calculated a relative feed conversion efficiency factor for each animal across groups. With the use of a mediation analysis approach, feed efficiency results were consistent with it being a significant mediator of longevity following the initial weight loss and stability phase (from 57‐83 weeks of age) for all groups, indicating that less efficient animals lived significantly longer **(**Table 2). This difference in feed efficiency was present even within groups being provided a daily ration of food (OWL, OWLM; Table 2).

**Table 2:** Mediation analyses

**Variable**	**Duration**	**Group**	**τ**	**τ'**	**α**	**SE α**	**β**	**SE β**	**Sobel test**	
									***t***	***P***
**Feed efficiency**	Week 57‐73	OWL	143.97	135.23	−0.07	0.08	42.86	12.62	2.51	**0.012**
		OWLM	96.63	72.58	0.04	0.07	42.86	12.62	8.00	**0.000**
		WC	78.80	11.47	1.01	0.07	42.86	12.62	5.15	**0.000**
	Week 73‐85	OWL	143.97	70.55	0.56	0.18	39.63	5.54	9.31	**0.000**
		OWLM	96.63	43.60	0.37	0.17	39.63	5.54	7.67	**0.000**
		WC	78.80	119.22	−1.74	0.17	39.63	5.54	−3.46	**0.001**
**Percent body fat**	Week 57‐ 73	OWL	143.97	159.12	−8.74	0.78	3.07	1.28	−1.32	0.186
		OWLM	96.63	78.61	−1.40	0.69	3.07	1.28	6.52	**0.000**
		WC	78.80	64.91	−3.30	0.68	3.07	1.28	2.95	**0.003**
	Week 73‐ 85	OWL	143.97	134.64	−8.94	0.98	4.70	1.07	0.88	0.380
		OWLM	96.63	63.17	−1.04	0.89	4.70	1.07	7.72	**0.000**
		WC	78.80	67.63	−3.69	0.89	4.70	1.07	1.94	0.053
**Fat:lean**	Week 57‐ 73	OWL	143.97	155.30	−0.28	0.02	82.42	41.86	−0.96	0.339
		OWLM	96.63	78.79	−0.05	0.02	82.42	41.86	6.23	**0.000**
		WC	78.80	64.78	−0.12	0.02	82.42	41.86	2.62	**0.009**
	Week 73‐ 85	OWL	143.97	135.38	−0.29	0.03	149.34	35.50	0.77	0.439
		OWLM	96.63	64.75	−0.04	0.03	149.34	35.50	7.40	**0.000**
		WC	78.80	71.13	−0.14	0.03	149.34	35.50	1.20	0.231
Sobel test (2‐sided)										

Using all animals without censoring; wave 1 and 2 combined.

To further explore these benefits of CR on longevity, we examined body composition outcomes. Adipose mass results were consistent with it being a significant mediator of the longevity benefit across groups, with lower body fat (mass, percent body fat, and fat to lean ratio) associated with longer life‐span (Figure 1 and 2; Table 1 and 2). Refining this adiposity measurement to include fat cell size, as measured in the subsample of collected mice that was dissected, showed that adipocyte cell size was significantly inversely related to group calorie intake (*P* < 0.001 inguinal and gonadal), consistent with the diet group by longevity relationship (Supporting Information Table S5 and S6; Supporting Information Figure S3).

There is a growing interest in the relationship between diet‐induced obesity with excess adiposity and inflammation, particularly related to the role of macrophage infiltration and senescent cells present in adipose depots. Inflammation is a prevalent condition and a potential contributor to multiple chronic and age‐related diseases. We therefore asked whether levels of inflammatory cytokines might explain the relationship between CR and life‐span. Circulating levels of serum cytokines were mostly unchanged among dietary groups at terminal collection (Supporting Information Table S9). In addition, we found no consistently different adipose‐tissue‐specific cytokine mRNA expression levels among the groups within the inguinal and gonadal adipose depots by quantitative PCR across the ages measured (Supporting Information Table S4). However, the lack of concordance between the adipose mRNA expression and circulating levels suggests an alternative source of production.

## Discussion

In both male and female mice, contrary to the epidemiologic literature, weight cycling resulted in longevity gains relative to having overweight or obesity and never intentionally losing weight. These gains in longevity were intermediate to achieving a normal body weight by CR and sustaining that restriction over the course of life, which has proven clinically difficult. However, the longevity benefit of weight cycling was similar to that of losing an intermediate amount of weight and sustaining the loss by chronic daily CR. While previous observational reports have highlighted the potential health benefits of weight reduction for individuals with overweight or obesity, unintentional weight regain following successful weight loss is a reality for the majority of persons with obesity losing weight. The results from the present study highlight that periods of CR and weight loss, even if dietary relapse occurs with weight regain, are superior to sustained excess weight and chronic adipose maintenance.

This study utilized a randomized controlled feeding design starting with a young adult cohort with high‐fat diet‐induced obesity, in which the weight cycling was achieved by means of energy restriction rather than alterations in dietary composition to model the modern human environment of excess caloric availability (often from sugar and fat) and sedentary lifestyle. Our results reinforce the significance of controlling energy intake within the context of a singular diet composition for health and longevity. Additionally, the large sample size provides robust power to investigate these effects on longevity and outcomes related to body weight, body composition, energy intake, and physiologic mediators of the longevity response. These outcomes were carefully measured along with proper controls (e.g., animals that were allowed to continue to *ad libitum* feeding and gain weight over the course of life), a comparison group that is often absent or not available for observational studies. This is particularly interesting considering the differential effects on longevity that we recently reported of prescribed energy intake (randomized at the group level) versus voluntary intake (observational at the individual level) (15).

Human weight loss is often accompanied by changes in diet composition and activity or exercise, as well as the use of pharmaceutical interventions, but has yet to be tested in a randomized experimental design for interactions related to long‐term health and longevity with weight cycling. The mediation analysis in the present study revealed a significant effect of feed efficiency and body fat on overall health and, ultimately, longevity outcomes. Although adipocyte cell size across treatment groups was related to longevity outcomes, in addition to the group food intake by longevity relationship, a conventional mediation analysis was not possible because the measures of adipocyte cell size were terminal assessments in only a subsample of mice. More directed tests of adipose‐related hormones, adipokines, or inflammatory markers were unable to identify a consistent biomarker or signature of longevity. The lack of a strong inflammatory signature contrasts with previous reports of high‐fat diet‐induced changes. The age of the mice at the time of measures, the sustained diet effects versus more acute changes following switching diet type, comparisons among groups fed only a high‐fat diet composition, and the smaller sample size of mice collected for these terminal measures are potential differences relative to previous reports (22, 23, 24, 25, 26).

One surprising observation during this study was the high number of animals that developed ulcerative dermatitis. Although ulcerative dermatitis is not uncommon in this mouse strain (C57BL/6), the duration of the study encompassing the entire life‐span and the diet composition may have contributed to the higher incidence, as published reports have noted risks with increasing age and high‐fat diet feeding in contrast to a lower incidence with “calorie restriction” (fed a low‐fat diet) (16, 27, 28). Despite this level of ulcerative dermatitis, the cause of death‐specific mortality analyses agreed with the overall group differences in survival.

Our findings demonstrating a decreased mortality rate with sustained CR or weight cycling relative to remaining with obesity are solidly supported. To the extent to which these findings represent those that might occur in humans, our results suggest that persons with obesity may benefit from weight loss in terms of longevity, even if the lost weight is regained and the loss‐gain cycle is repeated multiple times. If so, perhaps weight loss is not so ill‐fated a resolution after all (9).

## Funding agencies

This study was supported in part by NIH grant awards to DBA and TRN from R01AG033682, P30DK056336, P60DK079626, T32DK062710, U24AG056053, and P30AG050886. The opinions expressed are those of the authors and do not necessarily represent those of the NIH or any other organization.

## Disclosure

DBA reports grants from NIH during the conduct of the study and personal fees from Weight Watchers, personal fees from Medifast, and grants from Herbalife outside the submitted work. The other authors declared no conflict of interest.

## Author contributions

DBA, TRN, and DLS conceived, designed, and supervised the study; YY, YZ, JRV, and DLS assisted with data collection and interpretation. YY, AP, and SLD performed statistical analyses under supervision of DBA and TRN. DLS and DBA prepared initial versions of the manuscript, with all authors providing input and final approval of the submitted and published versions.
