# Supplementary material for: Weight Cycling Increases Longevity Compared with Sustained Obesity in Mice
Source: Obesity (Silver Spring). 2018 Oct 25;26(11):1733–9. doi: 10.1002/oby.22290 (PMC6221135; doi:10.1002/oby.22290)
Supplement: Supplementary file 1 [file OBY-26-1733-s001.pdf]

Figure S1. Body composition. Body composition as measured by QMR (mean±SD) post-randomization by group including wave 1 female (A, D, G) and male (B, E, H), and wave 2 male (C, F, I). Body fat mass as measured by QMR at subsequent loss cycle (post-randomization wk ~41; A-C; p<0.01 all groups vs. EO except (C) OWLM vs. EO, p=0.76) and lean mass following initial weight loss (T1 wk~13; D-F; p<0.01 all groups vs. EO) and regain by the WC group (T2 wk~29; D-F inset; p<0.01 except (D) inset EO vs. WC, p=0.923) and subsequent loss cycle (post-randomization wk ~41; G-I; p<0.01 all groups vs. EO) EO=Ever Obese (black), OWLM=Obese Weight Losers Moderate (red), OWL=Obese Weight Losers (blue), WC=Weight Cyclers (yellow).

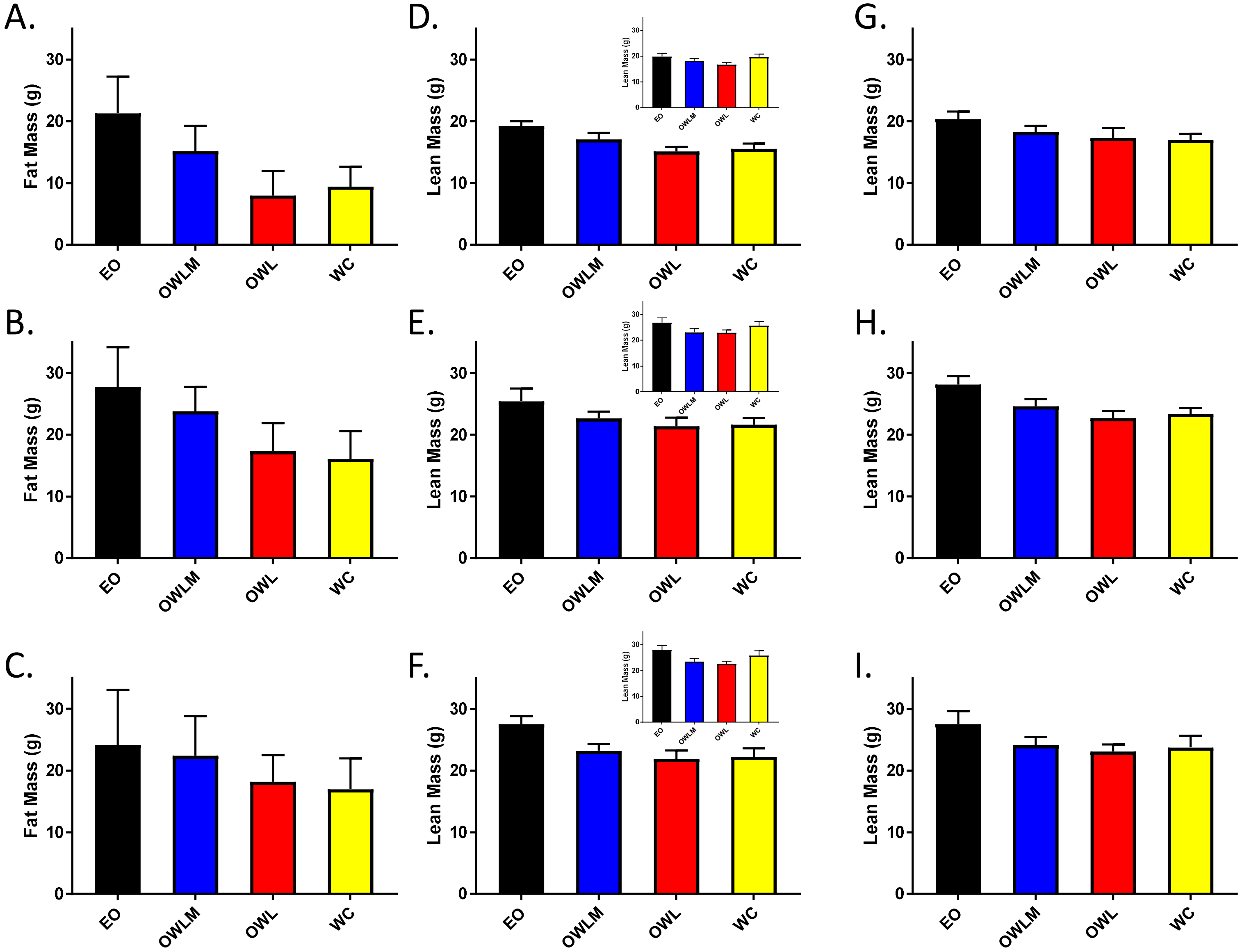

Figure S2. Adipose depot mass measured at dissection. Depot specific mass (mean±SD) for individual fat pads by sex (male: A,C; female: B,D) and age (initial weight loss: A,B, 50% survival: C,D) as measured at time of dissection. For analyses of significance among groups see **Table S2.**

Supplemental Figure 2

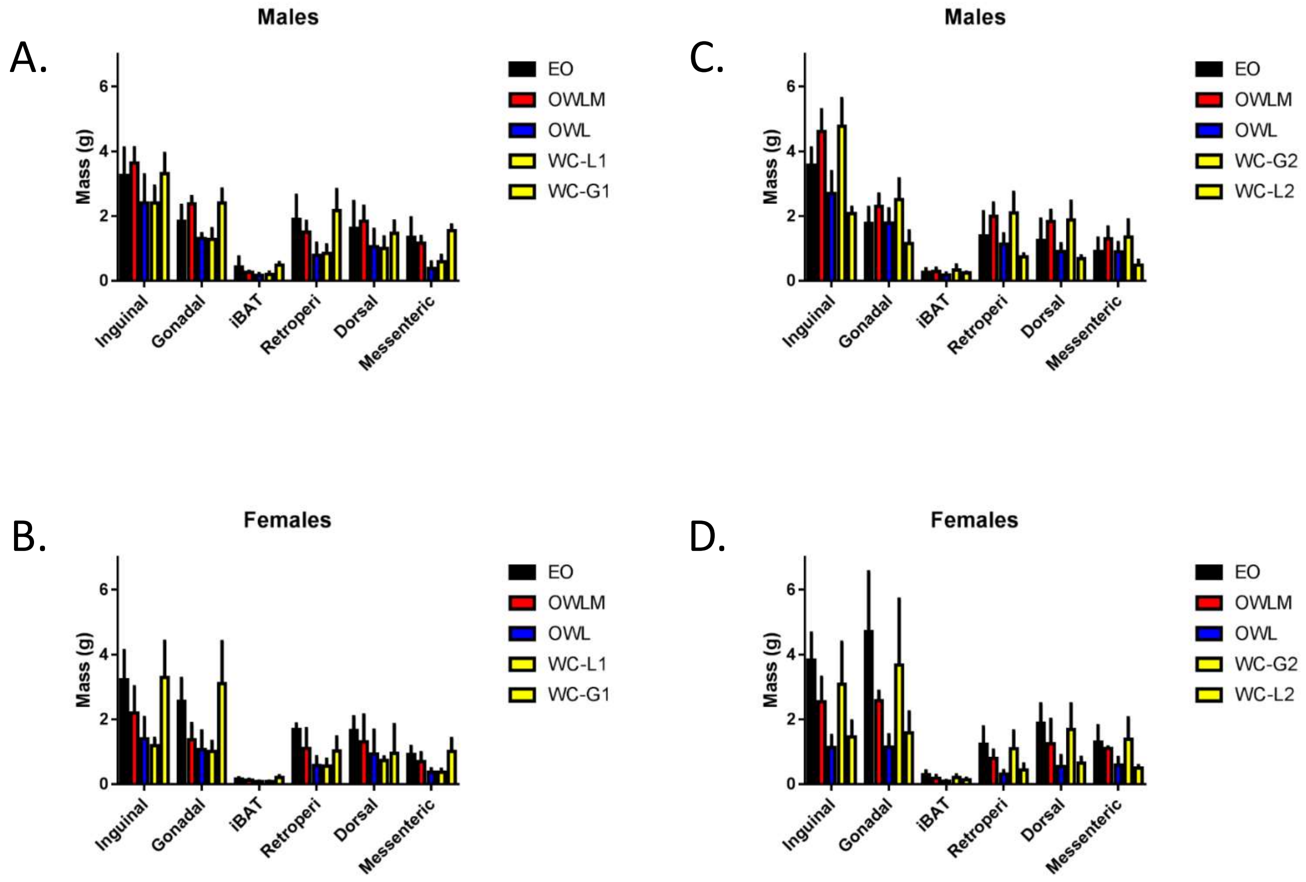

Figure S3. . Adipocyte cell size as measured by osmium tetroxide fixation. Adipocyte cell size (mean±SD) for gonadal (A-B, E-F) or inguinal (C-D, G-H) depots presented by sex (female-A, C, E, G and male- B, D, F, H) after initial weight loss (T1 – 1st) or mid-life collections (T3 – 2nd). Weight loss or regain by WC group shown (WC-L or WC-G; T2 and T4). Statistical results for group comparisons versus EO within a tissue at the specified time point (males and females combined) using GLM by group, adjusted for sex.

Figure S3

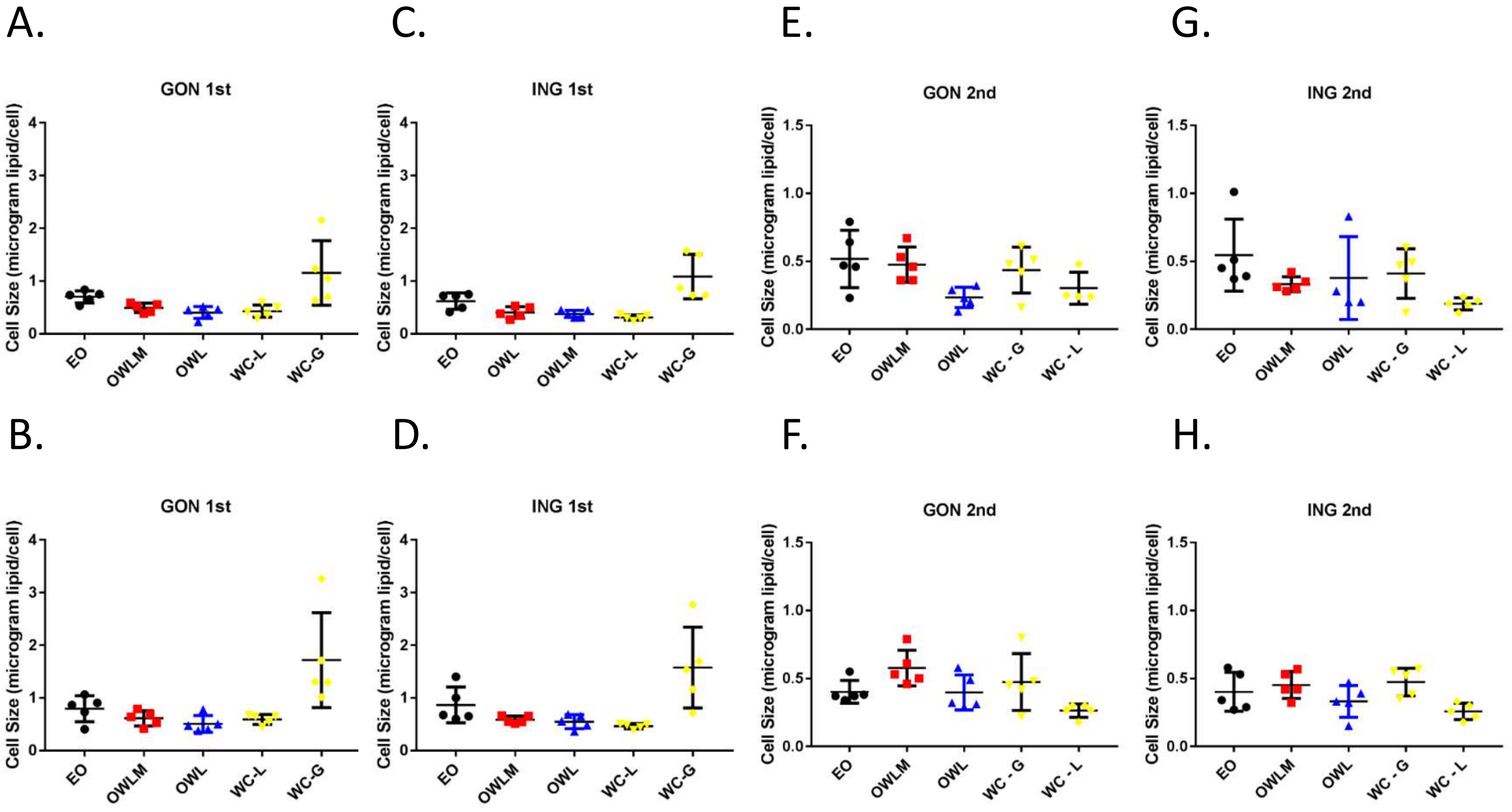

Comparison (EO vs.)	t-statistic	p-value	Tukey adjusted
OWLM	-3.15	**0.003**	**0.017**
OWL	-4.75	**<.001**	**<.001**
WC	-3.86	**0.001**	**0.003**

Comparison (EO vs.)	t-statistic	p-value	Tukey adjusted
OWLM	-4.01	**<.001**	**0.002**
OWL	-4.08	**<.001**	**0.001**
WC	-5.47	**<.001**	**<.001**

Comparison (EO vs.)	t-statistic	p-value	Tukey adjusted
OWLM	0.97	0.337	0.765
OWL	-2.09	**0.044**	0.175
WC	-0.07	0.943	0.999

Comparison (EO vs.)	t-statistic	p-value	Tukey adjusted
OWLM	-1.06	0.298	0.718
OWL	-1.53	0.136	0.433
WC	-0.41	0.683	0.976
