# Supplementary material for: Weight Cycling Increases Longevity Compared with Sustained Obesity in Mice
Source: Obesity (Silver Spring). 2018 Oct 25;26(11):1733–9. doi: 10.1002/oby.22290 (PMC6221135; doi:10.1002/oby.22290)
Supplement: Supplementary file 2 [file OBY-26-1733-s002.pdf]

Table S1. Diet Composition

	D12450B[#¥]		D12451[#¥]		D11022101[#¥†]		D11112301[#¥§]	
	gm%	*kcal%*	gm%	*kcal%*	gm	*kcal*	gm%	*kcal%*
Protein	19.2	*20.0*	23.7	*20.0*	22	*20*	23.7	*20.0*
Carbohydrate	67.3	*70.0*	41.4	*35.1*	40	*35*	40.3	*34.1*
Fat	4.3	*10.0*	23.6	*44.9*	22	*45*	23.6	*44.9*
Total		*100.0*		*100.0*		*100*		*99.0*
kcal/gm	3.85		4.73		4.5		4.73	

**Ingredient**	**gm**	***kcal***	**gm**	***kcal***	**gm**	***kcal***	**gm**	***kcal***
Casein, 80 Mesh	200	*800*	200	*800*	200	*800*	200	*800*
L-Cystine	3	*12*	3	*12*	3	*12*	3	*12*
Corn Starch	315	*1260*	72.8	*291.2*	72.8	*291*	72.8	*291.2*
Maltodextrin 10	35	*140*	100	*400*	100	*400*	100	*400*
Sucrose	350	*1400*	172.8	*691.2*	172.8	*691*	172.8	*691.2*
Cellulose	50	*0*	50	*0*	50	*0*	50	*0*
Soybean Oil	25	*225*	25	*225*	25	*225*	25	*225*
Lard	20	*180*	177.5	*1597.5*	177.5	*1598*	177.5	*1597.5*
Mineral Mix, S10026	10	*0*	10	*0*	0	*0*	10	*0*
Mineral Mix S10026B (50 gm/kg)					83.5	*0*		
DiCalcium Phosphate	13	*0*	13	*0*	0	*0*	13	*0*
Calcium Carbonate	5.5	*0*	5.5	*0*	0	*0*	5.5	*0*
Potassium Citrate, 1 H2O	16.5	*0*	16.5	*0*	0	*0*	16.5	*0*
Vitamin Mix, V10001	10	*40*	10	*40*	16.7	*67*	0	*0*
V13001 (w/o Vit A)	0	*0*	0	*0*			10	*40*
Vitamin A Acetate (500,000 IU/gm)	0	*0*	0	*0*			0.0035	*0*
Choline Bitartrate	2	*0*	2	*0*	2	*0*	2	*0*
FD&C Yellow Dye #5	0.05	*0*	0	*0*	0	*0*	0.025	*0*
FD&C Red Dye #40	0	*0*	0.05	*0*	0	*0*	0	*0*
FD&C Blue Dye #1	0	*0*	0	*0*	0.05	*0*	0.025	*0*
**Total**	**1055.05**	***4057***	**858.15**	***4057***	**903.35**	***4084***	**858.15**	***4057***
Vitamin A (IU/4057 kcals)	4074		4338		n.d.*		2088	
Vitamin A (IU/kg)	3861		5055		n.d.*		2433	

[#]Product Number

[¥]Research Diets, Inc., New Brunswick NJ, USA

[§]Rodent Diet With 45 kcal% Fat and Same With 67% Added Vitamins and Minerals

[†]Rodent Diet with 45 kcal% Fat and Reduced Vitamin A to NRC Value (2,400 IU / kg). Vitamin A calculated as 4,000 IU per 10 gm V10001, 168 IU / 100 gm Lard, and 20 IU / 100 gm Casein; Vitamin A should be assayed for accuracy.

*n.d. - not determined

Table S2. Adipose depot mass comparisons at specified time points.

Outcome	Time point	Comparison	p-value	FDR
Dorsal	1 & 2	all Groups	**0.019**	**0.033**
Dorsal	1 & 2	EO vs OWL	**0.020**	**0.034**
Dorsal	1 & 2	EO vs OWLM	0.811	0.860
Dorsal	1 & 2	EO vs WC_G1	0.117	0.164
Dorsal	1 & 2	EO vs WC_L1	**0.006**	**0.011**
Dorsal	3 & 4	all Groups	**<0.001**	**<0.001**
Dorsal	3 & 4	EO vs OWL	**0.001**	**0.003**
Dorsal	3 & 4	EO vs OWLM	0.911	0.911
Dorsal	3 & 4	EO vs WC_G2	0.369	0.445
Dorsal	3 & 4	EO vs WC_L2	**0.001**	**0.002**
Gonadal	1 & 2	all Groups	**<0.001**	**<0.001**
Gonadal	1 & 2	EO vs OWL	**0.001**	**0.003**
Gonadal	1 & 2	EO vs OWLM	0.289	0.367
Gonadal	1 & 2	EO vs WC_G1	0.065	0.097
Gonadal	1 & 2	EO vs WC_L1	**0.001**	**0.002**
Gonadal	3 & 4	all Groups	**0.001**	**0.001**
Gonadal	3 & 4	EO vs OWL	**0.001**	**0.002**
Gonadal	3 & 4	EO vs OWLM	0.117	0.167
Gonadal	3 & 4	EO vs WC_G2	0.774	0.834
Gonadal	3 & 4	EO vs WC_L2	**0.001**	**0.002**
iBAT	1 & 2	all Groups	**<0.001**	**<0.001**
iBAT	1 & 2	EO vs OWL	**0.001**	**0.003**
iBAT	1 & 2	EO vs OWLM	0.055	0.086
iBAT	1 & 2	EO vs WC_G1	0.310	0.388
iBAT	1 & 2	EO vs WC_L1	**0.003**	**0.007**
iBAT	3 & 4	all Groups	**0.012**	**0.021**
iBAT	3 & 4	EO vs OWL	**0.002**	**0.005**
iBAT	3 & 4	EO vs OWLM	0.411	0.479
iBAT	3 & 4	EO vs WC_G2	0.888	0.901
iBAT	3 & 4	EO vs WC_L2	0.060	0.092
Inguinal	1 & 2	all Groups	**<0.001**	**<0.001**
Inguinal	1 & 2	EO vs OWL	**<0.001**	**0.001**
Inguinal	1 & 2	EO vs OWLM	0.372	0.441
Inguinal	1 & 2	EO vs WC_G1	0.859	0.884
Inguinal	1 & 2	EO vs WC_L1	**<0.001**	**0.001**
Inguinal	3 & 4	all Groups	**<0.001**	**<0.001**
Inguinal	3 & 4	EO vs OWL	**<0.001**	**<0.001**
Inguinal	3 & 4	EO vs OWLM	0.743	0.813
Inguinal	3 & 4	EO vs WC_G2	0.532	0.611
Inguinal	3 & 4	EO vs WC_L2	**<0.001**	**<0.001**
Messenteric	1 & 2	all Groups	**<0.001**	**<0.001**
Messenteric	1 & 2	EO vs OWL	**<0.001**	**<0.001**
Messenteric	1 & 2	EO vs OWLM	0.150	0.202
Messenteric	1 & 2	EO vs WC_G1	0.237	0.307
Messenteric	1 & 2	EO vs WC_L1	**<0.001**	**<0.001**
Messenteric	3 & 4	all Groups	**<0.001**	**<0.001**
Messenteric	3 & 4	EO vs OWL	**0.047**	0.076
Messenteric	3 & 4	EO vs OWLM	0.585	0.661
Messenteric	3 & 4	EO vs WC_G2	0.136	0.186
Messenteric	3 & 4	EO vs WC_L2	**0.001**	**0.002**
Retroperi	1 & 2	all Groups	**<0.001**	**<0.001**
Retroperi	1 & 2	EO vs OWL	**<0.001**	**<0.001**
Retroperi	1 & 2	EO vs OWLM	**0.023**	**0.038**
Retroperi	1 & 2	EO vs WC_G1	0.339	0.417
Retroperi	1 & 2	EO vs WC_L1	**<0.001**	**<0.001**
Retroperi	3 & 4	all Groups	**<0.001**	**<0.001**
Retroperi	3 & 4	EO vs OWL	**0.007**	**0.014**
Retroperi	3 & 4	EO vs OWLM	0.686	0.762
Retroperi	3 & 4	EO vs WC_G2	0.183	0.241
Retroperi	3 & 4	EO vs WC_L2	**0.001**	**0.003**

*Timepoint: T1=initial weight loss (WC-L1), T2=initial WC regain (WC-G1), T3=50% survival (~2 yrs of age, WC-G2), T4=subsequent WC weight loss (WC-L2)

Table S3. Glucose and Hormones

		Group		Sex	
	**Timepoint***	**F-value**	**p-value**	**F-value**	**p-value**
Glucose	1	2.50	0.075	7.54	**0.010**
	3	0.08	0.972	0.45	0.505
Insulin†	1	8.96	**<0.001**	20.11	**<0.001**
	3	2.33	0.091	0.87	0.356
Adiponectin	1	2.05	0.124	82.30	**<0.001**
	3	0.62	0.604	19.57	**<0.001**
Leptin	1	10.15	**<0.001**	6.66	**0.014**
	3	2.90	**0.049**	0.10	0.748

*Timepoint: T1=initial weight loss, T3= 50% survival (~2 yrs of age)

†Insulin was log transformed before analysis to satisfy assumptions of Normality, with maximum level of detection at 10 ng/ml used for any value greater than the level of quantification.

Table S4. Depot Specific Adipokine and Inflammatory Markers

Variable (mRNA AU)§	Tissue	Timepoint	F-statistic	p-value	FDR
Adiponectin	GONADAL	T1	1.18	0.330	0.496
		T3	2.88	**0.049**	0.157
	INGUINAL	T1	3.32	**0.031**	0.151
		T3	5.53	**0.003**	0.058
Leptin	GONADAL	T1	4.27	**0.011**	0.094
		T3	2.47	0.078	0.202
	INGUINAL	T1	5.12	**0.005**	0.065
		T3	4.73	**0.007**	0.077
CD68	GONADAL	T1	3.48	**0.026**	0.156
		T3	1.01	0.398	0.544
	INGUINAL	T1	0.53	0.668	0.744
		T3	2.27	0.098	0.225
IL_6	GONADAL	T1	1.65	0.196	0.335
		T3	1.74	0.177	0.336
	INGUINAL	T1	0.48	0.696	0.744
		T3	1.92	0.145	0.319
PAI_1	GONADAL	T1	0.28	0.841	0.872
		T3	2.32	0.093	0.208
	INGUINAL	T1	1.17	0.335	0.488
		T3	1.99	0.134	0.288
Ppar	GONADAL	T1	0.55	0.652	0.734
		T3	1.79	0.167	0.327
	INGUINAL	T1	3.92	**0.016**	0.126
		T3	5.06	**0.005**	0.062
TGFb1	GONADAL	T1	2.34	0.090	0.221
		T3	1.36	0.270	0.411
	INGUINAL	T1	3.07	**0.040**	0.156
		T3	3.50	**0.025**	0.162
TNFalpha	GONADAL	T1	1.73	0.179	0.328
		T3	3.68	**0.021**	0.142
	INGUINAL	T1	2.63	0.065	0.196
		T3	2.48	0.077	0.203
Ppia	GONADAL	T1	1.07	0.374	0.518
		T3	0.21	0.885	0.894
	INGUINAL	T1	3.03	**0.042**	0.157
		T3	0.71	0.551	0.676

§mRNA AU - messenger RNA normalized to single gene, reported as arbitrary units (AU)
*Timepoint: T1=initial weight loss, T2=initial WC regain, T3=50% survival (~2 yrs of age), T4=subsequent WC weight loss

Table S5. Lifespan summary statistics (in days) by sex and diet group.

Gender	Group	N	mean	std	median	min	max
Male	EO	66	644.8	143.4	634.5	384	958
	OWLM	132	735.3	164.5	707.5	367	1151
	OWL	66	792.9	167.2	787.0	340	1047
	WC	138	703.8	147.6	702.0	402	1086
Female	EO	25	610.5	134.7	588.0	425	952
	OWLM	48	723.0	158.6	723.0	385	1050
	OWL	26	744.8	204.4	741.5	322	1126
	WC	51	742.2	179.6	725.0	343	1049
Males & Female	EO	91	635.4	141.2	630.0	384	958
	OWLM	180	732.0	162.6	711.0	367	1151
	OWL	92	779.3	178.7	778.5	322	1126
	WC	189	714.2	157.3	705.0	343	1086
Males & Female	All	552	717.86	165.48	705.0	322	1151

Table S6. Survival Analysis (All Cause Mortality)

data	predictor	Linear model		Survival model (Cox PH)	
		F-stat	p-value	HR	p-value
wave 1	**group**	5.54	**0.0010**		**< 0.001**
	**EO vs OWL**	3.65 *	**0.002 †**	0.44	**< 0.001**
	**EO vs OWLM**	3.52 *	**0.003 †**	0.53	**< 0.001**
	**EO vs WC**	2.43 *	**0.092 †**	0.63	**0.0090**
	**OWLM vs WC**	1.41 *	0.666 †	0.82	0.186
wave2	**group**	9.24	**< 0.001**		**< 0.001**
	**EO vs OWL**	5.26 *	**< 0.001 †**	0.32	**< 0.001**
	**EO vs OWLM**	3.13 *	**0.012 †**	0.49	**< 0.001**
	**EO vs WC**	3.2 *	**0.009 †**	0.52	**< 0.001**
	**OWLM vs WC**	0.1 *	1.000 †	0.93	0.6450

Using all animals without censoring

† corrected for multiple comparison using Sidak

**** t-statistic***

Table S7. Survival Analysis Considering Cause of Death with Censoring

data	predictor	Linear model		survival model (Cox PH)	
		F-stat	p-value	HR	p-value
**Model 1 - Technical complications censored**					
**wave 1**	**group**	6.10	**<0.001**		**<0.001**
	**EO vs OWL**	3.88*	**<0.001**	0.43	**<0.001**
	**EO vs OWLM**	3.67*	**<0.001**	0.52	**<0.001**
	**EO vs WC**	2.72*	**0.007**	0.59	**0.003**
**wave2**	**group**	9.31	**<0.001**		**<0.001**
	**EO vs OWL**	5.28*	**<0.001**	0.32	**<0.001**
	**EO vs OWLM**	3.24*	**0.001**	0.48	**<0.001**
	**EO vs WC**	3.26*	**0.001**	0.51	**<0.001**
**wave 1&2**	**group**	14.00	**<0.001**		**<0.001**
	**EO vs OWL**	6.29*	**<0.001**	0.37	**<0.001**
	**EO vs OWLM**	4.88*	**<0.001**	0.50	**<0.001**
	**EO vs WC**	4.17*	**<0.001**	0.55	**<0.001**

Model 2 - Technical complications and euthanized for reasons other than ulcerative dermatitis censored

**wave 1**	**group**	5.81	**<0.001**		**<0.001**
	**EO vs OWL**	3.54*	**<0.001**	0.41	**<0.001**
	**EO vs OWLM**	3.47*	**<0.001**	0.56	**0.003**
	**EO vs WC**	1.84*	0.068	0.63	**0.015**
**wave2**	**group**	8.25	**<0.001**		**<0.001**
	**EO vs OWL**	4.95*	**<0.001**	0.31	**<0.001**
	**EO vs OWLM**	3.25*	**0.001**	0.49	**<0.001**
	**EO vs WC**	3.11*	**0.002**	0.49	**<0.001**
**wave 1&2**	**group**	12.74	**<0.001**		**<0.001**
	**EO vs OWL**	5.86*	**<0.001**	0.35	**<0.001**
	**EO vs OWLM**	4.76*	**<0.001**	0.52	**<0.001**
	**EO vs WC**	3.39*	**<0.001**	0.55	**<0.001**

Model 3 - All causes of death except 'found dead' censored

**wave 1**	**group**	2.43	0.070		0.14
	**EO vs OWL**	2.48*	**0.015**	0.43	**0.02**
	**EO vs OWLM**	2.35*	**0.021**	0.56	0.07
	**EO vs WC**	1.74*	0.085	0.62	0.14
**wave2**	**group**	4.37	**0.006**		**<0.001**
	**EO vs OWL**	3.42*	**<0.001**	0.18	**<0.001**
	**EO vs OWLM**	2.35*	**0.020**	0.34	**<0.001**
	**EO vs WC**	2.64*	**0.009**	0.28	**<0.001**
**wave 1&2**	**group**	6.16	**0.001**		**<0.001**
	**EO vs OWL**	4.08*	**<0.001**	0.26	**<0.001**
	**EO vs OWLM**	3.38*	**<0.001**	0.41	**<0.001**
	**EO vs WC**	3.01*	**0.003**	0.39	**<0.001**

**** t-statistic***

Table S8. Correlation between 'age at max weight' and lifespan.

Group	Sex	15 weeks post-randomization	p-value
EO	Male	0.85	<0.001
	Female	0.92	<0.001
	both	0.87	<0.001
OWL	Male	0.57	<0.001
	Female	0.48	0.024
	both	0.54	<0.001
OWLM	Male	0.75	<0.001
	Female	0.69	<0.001
	both	0.74	<0.001
WC	Male	0.77	<0.001
	Female	0.85	<0.001
	both	0.80	<0.001

Maximum weight was considered between 15 weeks post-randomization and observed death of an animal.

Table S9. Serum Cytokines.

Cytokine	Timepoint*	F-value	p-value	FDR
IFN_y	1	1.69	0.187	0.481
IFN_y	3	0.46	0.714	0.803
IL_10	1	2.41	0.083	0.748
IL_10	3	0.67	0.574	0.737
IL_1B	1	1.81	0.163	0.490
IL_1B	3	0.72	0.547	0.758
IL_2	1	1.04	0.387	0.775
IL_2	3	0.33	0.804	0.804
IL_5	1	1.96	0.138	0.620
IL_5	3	1.38	0.264	0.595
IL_6	1	1.94	0.141	0.508
IL_6	3	0.35	0.787	0.833
TNF_a	1	2.30	0.094	0.566
TNF_a	3	0.67	0.579	0.695
KC_GRO	1	5.60	**0.003**	**0.054**
KC_GRO	3	0.76	0.526	0.788

*Timepoint: T1=initial weight loss, T3= 50% survival (~2 yrs of age)

Table S10. Benchmark body weight of male and female C57BL/6J mice[¥] fed D12450B diet *ad libitum* .

Age (weeks)	Male		Female	
	Mean	SD	Mean	SD
51	34.68	3.51	24.32	1.37
55	35.86	3.85	24.89	1.62
59	35.95	3.64	25.10	1.66
63	36.61	3.38	25.40	1.51
67	38.03	3.36	26.07	1.67
71	39.22	3.73	26.20	2.35
75	40.15	3.81	27.17	2.64
79	40.19	4.10	27.44	3.12
83	40.26	3.73	28.11	3.32
87	40.93	4.26	27.65	3.29
91	39.81	5.26	27.67	2.96
95	39.57	4.70	28.73	3.49
99	38.25	4.17	27.75	2.75
103	39.14	4.37	27.87	3.78
107	38.32	4.71	28.84	5.16
111	38.43	2.85	30.36	5.63
115	35.82	3.10	29.92	5.01
119	36.45	4.09	29.25	5.13
123	33.25	4.63	28.16	5.06
127	34.45	2.41	27.64	4.61
131	32.56	n.a.	25.87	4.27

[¥] n=15/sex at study initiation
